# Effect of Aggregate Size on the Axial Compressive Behavior of FRP-Confined Coral Aggregate Concrete

**DOI:** 10.3390/polym14183877

**Published:** 2022-09-16

**Authors:** Pengda Li, Deqing Huang, Ruiyu Li, Rongkang Li, Fang Yuan

**Affiliations:** 1 College of Civil and Transportation Engineering, Shenzhen University, Shenzhen 518060, China; 2 Guangdong Provincial Key Laboratory of Durability for Marine Civil Engineering, Shenzhen University, Shenzhen 518060, China; 3 Key Laboratory for Resilient Infrastructures of Coastal Cities, Ministry of Education, Shenzhen University, Shenzhen 518060, China

**Keywords:** coral aggregate concrete, aggregate size, fiber-reinforced polymer, axial compression, confinement

## Abstract

Using locally available raw materials for preparing concrete, such as coral reefs, seawater, and sea sand, is conducive to compensating for the shortage of construction materials used on remote islands. Jacketing fiber-reinforced polymer (FRP), as passive confinement, is a practical approach to enhance the strength, ductility, and durability of such coral aggregate concrete (CAC). Rational and economical CAC structural design requires understanding the interactions between the CAC fracture process and FRP confinement. The coral aggregate size is the critical parameter of their interaction since it affects the crack propagation of CAC and FRP confinement efficiency. This study conducted axial compression tests on FRP-confined CAC cylinders with varying coral aggregate sizes and FRP confinement levels. The test results indicate that the coral aggregate sizes affected the unconfined CAC strength. In addition, the dilation behavior of FRP-confined CAC varied with aggregate sizes, showing that CAC with smaller coral aggregate featured a more uniform hoop strain distribution and larger FRP rupture strain. These coupling effects are epitomized by the variation in the transition stress on the stress–strain curve, which makes the existing stress–strain models not applicable for FRP-confined CAC. A modified stress–strain model is subsequently proposed. Finally, the practical and environmental implications of the present study are discussed.

## 1. Introduction

In the South China Sea, there are many coral islands and reefs in the ocean, which are coral polyp skeletons composed of thousands of tons of calcium carbonate. This coral debris washed ashore by seawater provides a new type of building material for engineering construction. Meanwhile, there is an extreme lack of sand, gravel, and freshwater resources on the islands hundreds or even thousands of kilometers away from the mainland. The transportation of these raw materials for concrete will greatly increase the cost of projects and extend construction periods due to the disturbance of natural weather conditions. On the premise of not destroying the natural ecosystem of coral reefs, it is of great significance to prepare the required concrete by using local rich coral aggregate resources [1,2].

In recent years, numerous studies have been devoted to investigating the properties of coral aggregate concrete (CAC) [3,4,5,6,7,8,9]. Compared to natural aggregates, coral aggregates exhibit lower density, rough surface texture, higher flakiness index values, and higher water absorption due to their low intensity, fragility, porous nature, and nonhomogeneity [3,4,5]. Owing to these unique characteristics of coral aggregate, CAC exhibits lower strength, more pronounced brittleness, and poorer workability than natural aggregate concrete (NAC) [6,7,8,9]. Da et al. [6] and Wang et al. [7] tested the uniaxial compressive behavior of CAC and found that it showed a more brittle failure mode than NAC. The post-peak stress decreased suddenly to 0.30–0.50 of the peak stress, and the enclosed area of the uniaxial compressive stress–strain curve of CAC was only half the enclosed area of the uniaxial compressive stress–strain curve of NAC. Zhou et al. [8] tested the uniaxial and triaxial compression behavior of CAC and proposed the corresponding constitutive relationships. Ma et al. [9] investigated the effect of the strain rate on the mechanical characteristics of CAC and found that the rate dependence on the compressive strength was more remarkable for CAC than for other cement-based composites. The obvious brittleness of CAC severely limits its application in engineering practices. The other major problem in the application of coral aggregate, seawater, and sea sand is that of durability. The rich chloride ions in these raw materials make them unsuitable for traditional steel-reinforced structures [10,11].

Fiber-reinforced polymers (FRPs) are attracting widespread attention in engineering practices because of their excellent strength and immunity to chloride-induced corrosion [12,13]. External jacketing using FRP sheets will put concrete in a state of triaxial compression, thereby greatly enhancing the mechanical properties of the concrete [14]. Recently, several studies have investigated the axial compressive properties of FRP-confined CAC [7,15,16]. Ying et al. [15] investigated the axial compressive behavior of glass FRP (GFRP)-confined CAC and compared it with that of GFRP-confined NAC. They found that the glass FRP (GFRP)- and GFRP-confined NAC exhibited similar failure modes, and the strength and deformation capacity of CAC could be significantly increased through FRP confinement. Wang et al. [7] and Zhang et al. [16] used prefabricated GFRP tubes to confine CAC and found an evident increase in ductility. The transition segment of FRP-confined CAC was lower than the transition segment of FRP-confined NAC. Actually, the difference in the mechanical properties between CAC made with coral aggregate, seawater, and sea sand and NAC prepared from natural gravel, freshwater, and river sand lies in the differences in the aggregates. Previous studies have indicated that the strength and working performance of seawater sea sand concrete are similar to the strength and working performance of freshwater river sand concrete, where the former exhibited slightly higher early compressive strength [17,18,19]. The mechanical performance of CAC is sensitive to the properties of coral aggregates, where the size of the aggregate should be the priority. Previous studies on the aggregate size effect of NAC indicated that the strength of unconfined CAC [20,21,22] as well as the stress–strain response and confinement efficiency of FRP-confined NAC were affected by the aggregate size [23,24]. However, there are few reports regarding the effect of coral aggregate size on the properties of CAC with or without FRP confinement.

This paper is an attempt to investigate the influence of aggregate size on the characteristics of FRP-confined CAC subjected to uniaxial compression. First, a group of FRP-confined CAC specimens with the test parameters of aggregate size and FRP thickness were prepared and tested. The experimental results, including the failure modes, axial stress versus axial/lateral strain response, lateral strain distribution, and ultimate conditions, were analyzed in detail. A modified model suitable for FRP-confined CAC was subsequently proposed based on the regression analysis of the acquired test results.

## 2. Experimental Program

### 2.1. Design and Preparation of the Specimens

A total of 27 cylinder specimens with a height (*H*) of 300 mm and diameter (*D*) of 150 mm were manufactured. The test variables included the coral aggregate size (*d_a_*) and the FRP thickness (*t_f_*). The test specimens consisted of three groups according to the continuous gradation of the coral aggregate involving 5–10 mm, 10–16 mm, and 16–26 mm. Each group of specimens was confined by 1-ply or 2-ply carbon FRP (CFRP) sheets. The unconfined cylinders were also prepared as control specimens for each group. Each test configuration included three identical nominal specimens. Table 1 gives detailed information on the designed specimens. The naming rules of the specimens are as follows: (1) the first two letters, CA, followed by a number, represent the coral aggregate size; (2) the letter P, followed by a single-digit number after the first hyphen, indicates the number of plies of FRP sheets; (3) the number after the second hyphen (1/2/3) differentiates the three nominal identical specimens. For example, CA10-P2-3 represents the third of the three nominally identical FRP-confined CAC cylinders with aggregate sizes of 10–16 mm and two-ply CFRP wraps.

The specimens of the same concrete mix were cast from the same batch of concrete. After 28 days of curing, the carbon fiber sheets were impregnated with the epoxy resin adhesive and wrapped gradually onto the specimen through a wet lay-up process, with an overlap region of 150 mm. The CFRP fibers were only oriented in a hoop direction to guarantee the maximum confinement efficiency. After 7 days of room temperature maintenance, the gypsum was placed on the cylinder to flush the concrete surface with the loading plate.

### 2.2. Material Properties

**Figure 1:**
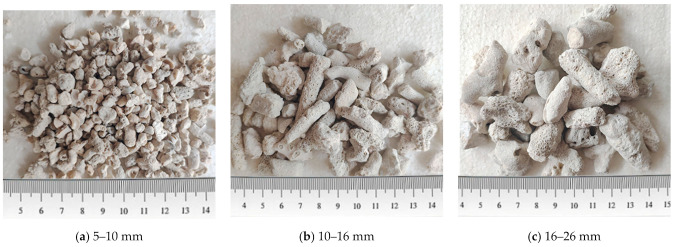
Coral aggregates of different sizes.

The materials used in the casting of the CAC consisted of ordinary Portland cement (P.0.42.5), coral aggregate, seawater, sea sand, and polycarboxylate superplasticizer. Table 2 shows the chemical composition of the seawater. The physical properties and chemical composition of the sea sand are shown in Table 3. The coral aggregate was purchased from Lingshou, Hebei Province, China, and originally produced in the Philippines. In order to filter the aggregates by size, standard sieves were used in this study, which were 26 mm (sieve hole), 16 mm, 10 mm, and 5 mm. Three kinds of coral aggregates with sizes between two adjacent sieves were obtained, which were 5–10 mm, 10–16 mm, and 16–26 mm (Figure 1). The properties of the coral aggregates were determined based on the Chinese standard GB/T 14685 [25], as shown in Table 4. Table 5 shows the mixture composition of the CACs with different aggregate sizes. Notably, due to the larger specific surface area and higher water absorption of coral aggregates with smaller sizes, the required amount of superplasticizer was relatively larger to ensure the same workability. Three cubes with the dimensions of 100 × 100 × 100 mm were cast for each type of CAC. The average compressive strengths (*f_cu_*) for the CA5, CA10, and CA16 concrete-mix cubes were 33.5 MPa, 32.9 MPa, and 27.2 MPa, respectively. The epoxy resin adhesive hardener and epoxy resin adhesive base resin were E2500S from Kony Bond, and the mixing weight ratio of the base resin and hardener was 2:1. The CFRP sheet possessed a thickness of 0.167 mm, produced by Toray Industries, Inc. Nihonbashi Muromachi, Chuo-ku, Tokyo. The fiber weight was 300 g/m^2^ and the real density was 1.80 g/cm^3^. The mechanical properties of the CFRP were determined by the average value of the five tensile coupon tests according to ASTM D3039M [26]. The results are summarized in Table 6.

### 2.3. Testing and Instrumentation

**Figure 2:**
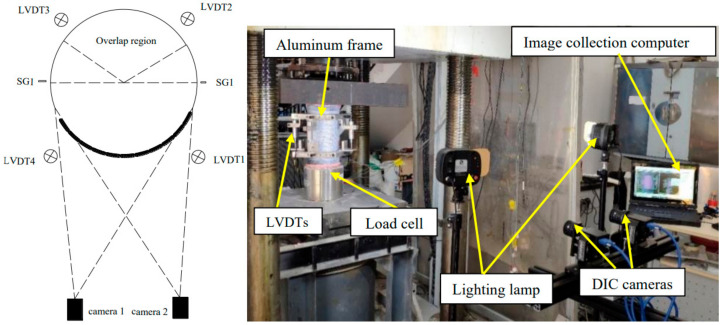
Test setups.

The load was applied through the load-based control option with a load rate from 2 kN/s to 250 kN followed by the displacement-based control option with a displacement rate of 0.3 mm/min until the failure of the specimens. Four linear variable displacement transformers (LVDTs), 90° apart along the circumference of the cylinder, were arranged to obtain the axial deformation/strain of the specimens. The measured length of the LVDTs was 185 mm, aligned along the axial direction. Two strain gauges (SGs) were symmetrically and vertically attached outside the overlapping region to measure the axial strains of the cylinders (SG1, SG2). The digital image correlation (DIC) systemproduced by Correlated Solutions, Inc. Irmo, USA was employed to measure the strain field on the specimen, which has proven to be highly accurate and effective for continuously capturing the strain fields during loading [27,28]. The test setup and instrumentations are shown in Figure 2. The measured data were recorded by a DEWESOFT dynamic acquisition system produced by Dewesoft, Trbovlje, Slovenija.

## 3. Experimental Results and Discussions

### 3.1. Failure Modes

**Figure 3:**
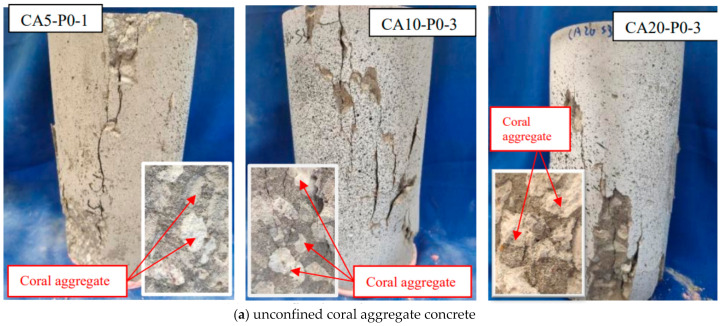

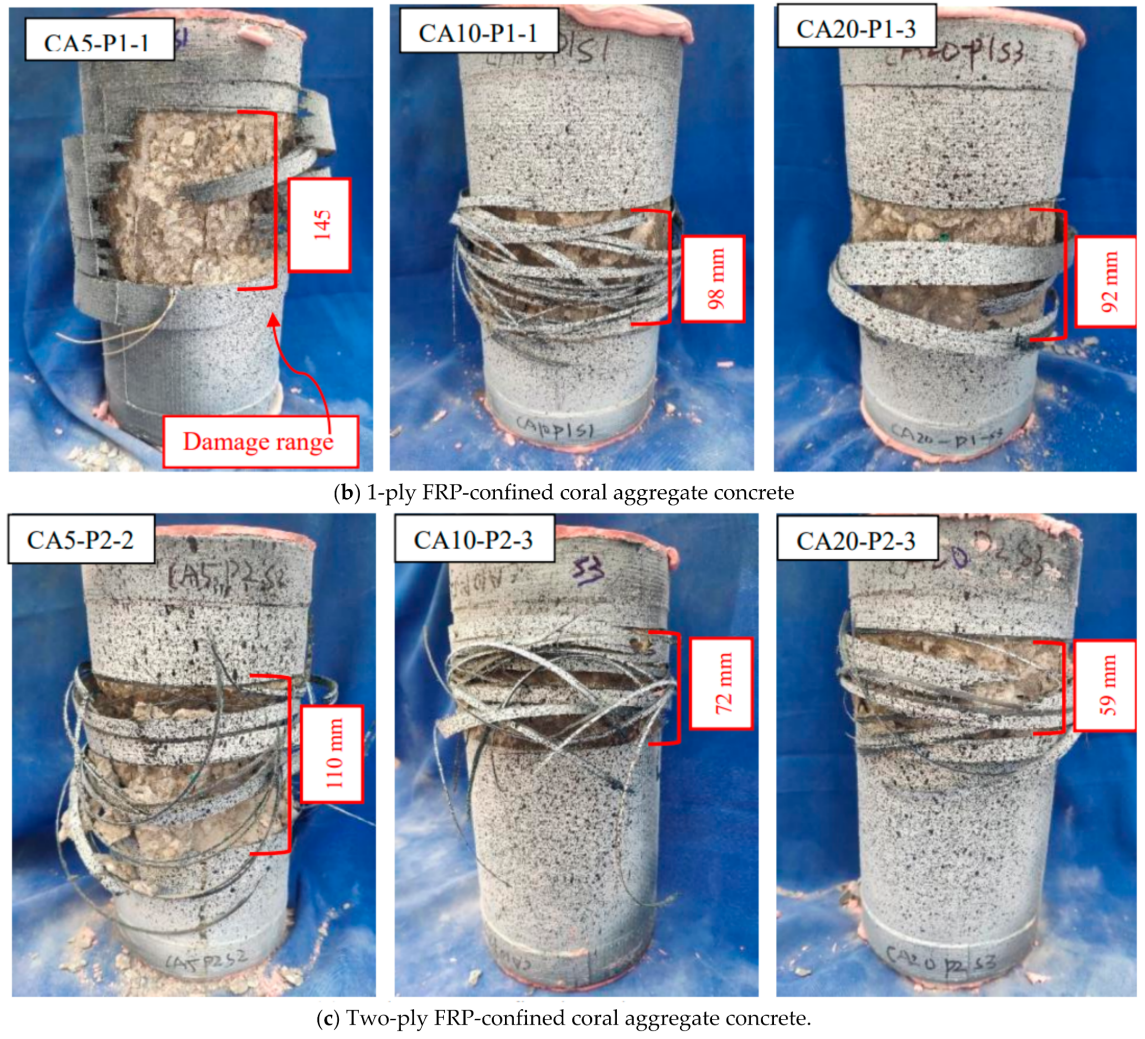
Failure patterns of the specimens.

The failure modes of the specimens are given in Figure 3. For the unconfined CAC cylinders, microcracks first appeared on the surface of the cylinders. With increasing loading, these microcracks gradually penetrated each other to form larger and longer cracks. The columns failed very suddenly when the peak strength was reached, accompanied by a crisp sound and a main oblique crack from the top to the bottom. The final failure pattern of the unconfined CAC is shown in Figure 3a. After the compressive test, the spalled CAC was collected for further examination. The cracked surfaces were observed to be smooth, and all the coral aggregates across the cracks were broken (Figure 3a), which is very different from the test observations for NAC, where the cracks always develop along the interface transition zone between the mortar and aggregate [10]. This difference is due to the much lower strength of coral aggregate compared to natural aggregate. As seen in Figure 3b,c, for the FRP-confined CAC, the failure was governed by FRP rupture at the column mid-height outside the overlapping zone. The height of the FRP rupture zone was greatly influenced by the coral aggregate size. Under the same level of FRP confinement, the greater the aggregate size of CAC was, the smaller the rupture area of the FRP sheet was. For example, the heights of the damage zone for CA5-P1-1 and CA5-P2-2 with aggregate sizes of 5–10 mm were 145 mm and 110 mm, respectively, which decreased to 92 mm and 59 mm for CA20-P1-3 and CA20-P2-3 when the aggregate size increased to 15–26 mm, suggesting that the coral aggregate size had a pronounced influence on the dilation of CAC. The CAC with a smaller aggregate size dilated more uniformly than the CAC with the same FRP confinement but a larger aggregate size.

### 3.2. Stress–Strain Behavior

**Figure 4:**
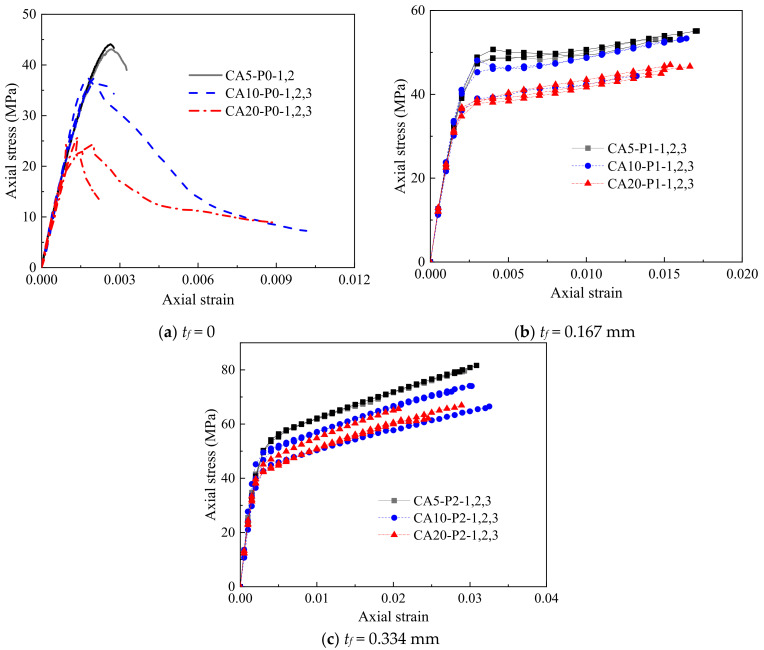
Effect of coral aggregate size on stress–strain behavior of confined CAC.

**Figure 5:**
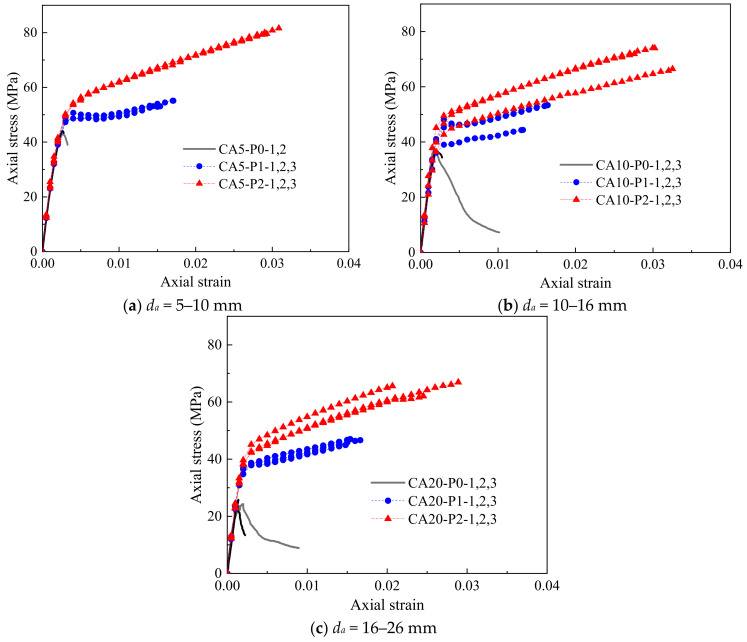
Effect of FRP thickness on stress–strain behavior of confined CAC.

The stress–strain curves of all FRP-confined CAC specimens are shown in Figure 4 and Figure 5. The strength and deformation indices of the specimens are summarized in Table 1, where *f_co_* = the compressive strength of the unconfined CAC, *ε_co_* = the compressive strain at the peak stress of the unconfined CAC, *f_cc_* = the ultimate axial strength, *ε_cc_* = the ultimate axial strain of the FRP-confined CAC, respectively, *E_c_* = the initial modulus of elasticity of the CAC, *E*_2_ = the slope of the linear second segment of the stress–strain curve, and *f*_0_ = the intercept of the stress axis by the linear second portion of the stress value at the intercept of the asymptotical line with the vertical axis. The enhancement ratios of the ultimate condition (i.e., *f_cc_*/*f_co_* and *ε_cc_*/*ε_co_*) are also listed in Table 1.

Figure 4 gives the effect of the coral aggregate size on the stress–strain curves of CAC. The axial stress was found to first increase almost linearly with increasing axial strain and then decrease rapidly beyond the peak stress for all unconfined CAC specimens with each aggregate size (Figure 4a), suggesting poor ductility for these specimens. The aggregate size had little effect on the elastic modulus but demonstrated a pronounced influence on the strength of the unconfined CAC. With a decreasing coral aggregate size from 16–26 mm to 5–10 mm, the average compressive strength increased from 24.9 MPa to 43.6 MPa, which was attributed to the difference in the apparent shapes of the coral aggregates with various sizes, as shown in Figure 1. The larger the aggregate size was, the slenderer the shape, and thus, the more likely and easier it was for the coral aggregate to be penetrated and split by cracks during compression. In contrast, aggregates with smaller sizes possessed a more rounded shape, and the strength of the CAC prepared with this kind of aggregate was higher. Figure 4b,c shows the effect of the aggregate size on the stress–strain curves of the FRP-confined CAC with the same FRP thickness. The stress–strain responses generally followed a bilinear feature with a transition zone in between. The ultimate stress and the value of *f*_0_ at the transition zone were significantly decreased with the increasing aggregate size. However, the aggregate size had nearly no effect on the initial and secondary slopes of the stress–strain response.

Figure 5 shows the effect of the FRP thickness on the stress–strain curves of the FRP-confined CAC with the same aggregate size. The first segment of the stress–strain curves of the FRP-confined CAC was almost identical to that of the unconfined CAC due to the small lateral dilation of CAC at this segment, while the slope of the second segment was governed by the degree of FRP confinement. The higher the degree of confinement was, the more rapid the increase in the axial stress was. For the 1-ply FRP-confined CAC, a minor reduction in stress was observed in the transition zone, which was not observed for the 2-ply FRP-confined CAC. This test observation was also reported by Wang et al. [7], who believed that this stress drop was attributed to the compacting process for CAC before its rapid dilation and the subsequent activation of FRP confinement.

### 3.3. Lateral Strain Distribution

**Figure 6:**
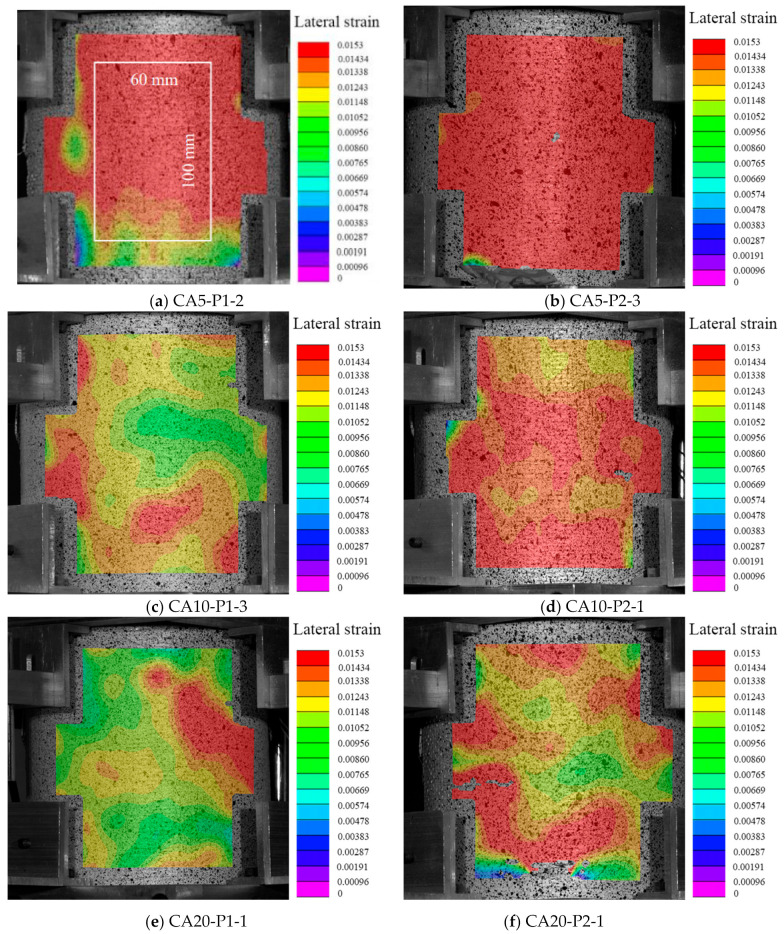
Lateral strain distributions.

**Figure 7:**
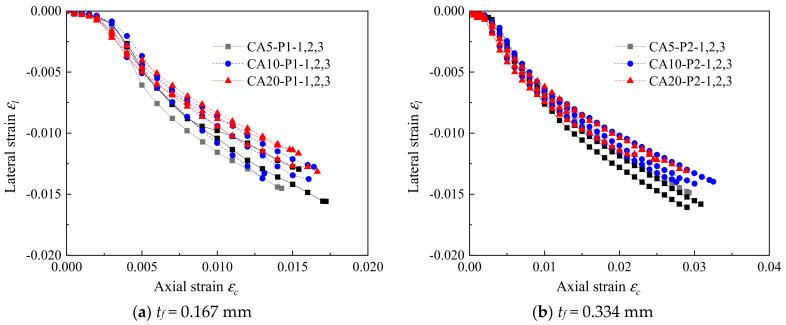
Effect of coral aggregate size on axial strain–lateral strain curves of confined CAC.

**Figure 8:**
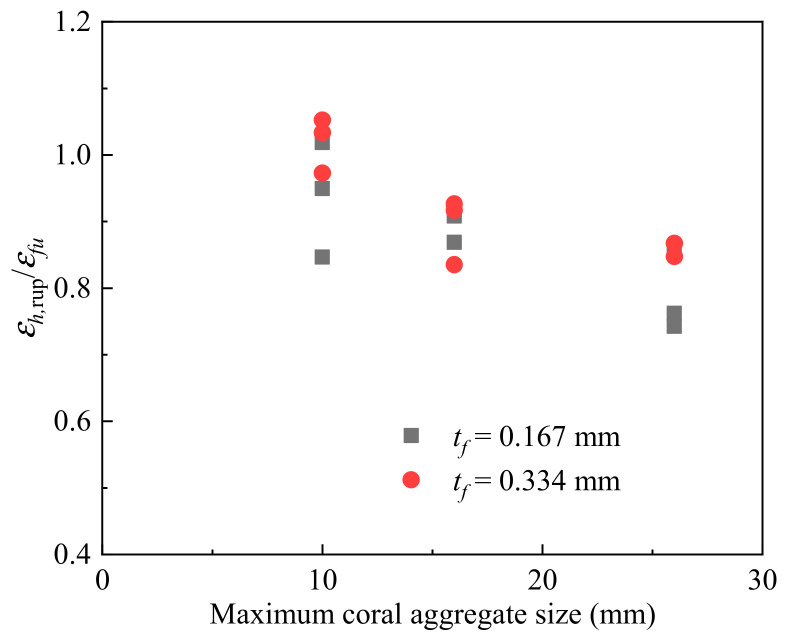
Effect of coral aggregate size on ultimate lateral strain.

The strain field on the specimen was collected and analyzed by a digital image correlation (DIC) system. The lateral strain distribution of the FRP-confined CAC at the final state is shown in Figure 6. For each group, two specimens with different numbers of FRP layers were selected as the representatives. The lateral strain was found to be distributed much more uniformly for the FRP-confined CAC with smaller coral aggregate sizes, indicating that a larger area of FRP reached its ultimate tensile strain at the ultimate state, resulting in a larger FRP rupture region, as shown in Figure 3b,c. The ratio of the ultimate FRP lateral strain acquired from the compression test (*ε_h,_*_rup_) to the ultimate tensile strain from the coupon test (*ε_fu_*) is shown in Figure 7. In this test, the *ε_h,_*_rup_ was achieved by the average lateral strain of the selected middle area of the test specimen (e.g., 100 mm long and 60 mm wide). A clear decreasing trend of the *ε_h,_*_rup_/*ε_fu_* was observed with increasing coral aggregate sizes, suggesting higher FRP confinement efficiency for specimens with smaller aggregate sizes (Figure 7). To further examine the dilation properties of CAC during the axial loading, the axial-to-lateral strain curves are given in Figure 8. Under the same lateral strain, a larger axial strain was generally observed for those specimens with a greater coral aggregate size, which can be described as follows: under the same confining pressure *σ_l_*, the confining pressure ratio *σ_l_*/*f_co_* was larger for specimens with a larger aggregate size since the compressive strength of the unconfined CAC *f_co_* decreased with the aggregate size. As a result, the larger FRP confinement stiffness ratio and the relatively “softening concrete” resulted in a larger axial strain.

### 3.4. The Ultimate Condition

**Figure 9:**
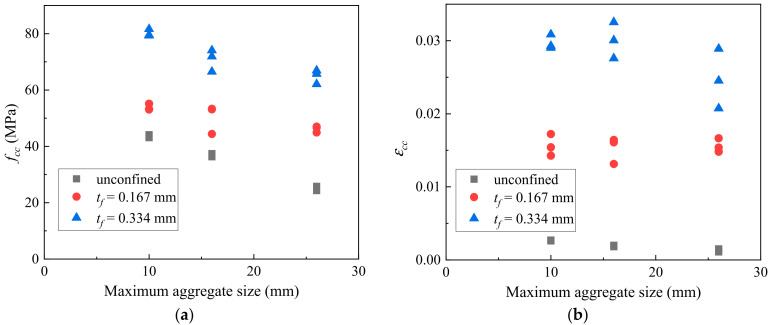
Effect of coral aggregate size on: (**a**) compressive strength; (**b**) ultimate axial strain.

The variations in the ultimate condition of CAC specimens with or without the external FRP confinement in terms of *f_cc_* and *ε_cc_* are shown in Figure 9. For the unconfined specimens, the strain at the compressive strength is used to represent *ε_cc_* rather than the actual ultimate axial strain, as the latter was difficult to obtain during the tests. Figure 9a shows that the *f_cc_* value decreased almost linearly with increasing aggregate sizes. The decreasing trend was similar for specimens under different degrees of FRP confinement, suggesting a negligible effect of the aggregate size on the strength enhancement of CAC achieved from FRP confinement. It is seen in Figure 9b that no clear variation trend of the ultimate axial strain of the FRP-confined CAC was observed with the increase in the aggregate size. The lateral dilation of the CAC was more pronounced for the confined CAC specimens with smaller aggregate sizes due to the more uniform distribution of the lateral strain. Moreover, the axial strain at the same confining pressure was smaller for those specimens with a smaller aggregate size, as explained in the preceding section. This resulted in an almost constant value of the ultimate axial strain of the FRP-confined CAC specimens except for those with an aggregate size of 16–26 mm and 2-ply FRP confinement.

**Figure 10:**
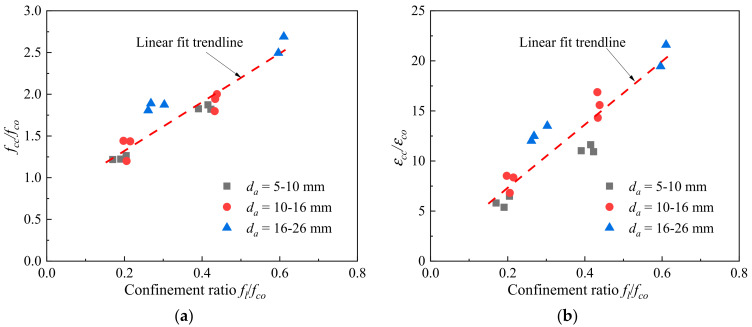
The strength enhancement ratio–confinement ratio relation and strain enhancement ratio–confinement ratio relation. (**a**) Ultimate strength enhancement ratio. (**b**) Ultimate strain enhancement ratio.

Figure 10 shows the strength enhancement ratio (*f_cc_*/*f_co_*) and strain enhancement ratio (*ε_cc_/ε_co_*) against the confinement ratio (*f_l_/f_co_*), in which *f_l_* is the confining pressure induced by the FRP sheet at failure, which can be expressed as

(1) $$f_{l}=\frac{2E_{f}t_{f}\varepsilon_{h,\mathrm{rup}}}{D}$$

where *E_f_* = the modulus of elasticity of the FRP, and *D* = the column diameter. Both the *f_cc_*/*f_co_* and *ε_cc_/ε_co_* increased with the increasing confinement ratio, and their variations followed a similar trend, as indicated in the linear fit trendlines in Figure 10, indicating that the coral aggregate size had little influence on the strength and strain enhancements as the confinement ratio increased.

## 4. Comparisons of the Available Stress–Strain Models

To evaluate the feasibility of the available stress–strain models of the FRP-confined concrete on the FRP-confined CAC with different aggregate sizes, the measured stress–strain curves were compared with the predicted results from several available stress–strain models given in previous studies: (i) the design-oriented model of FRP-confined NAC proposed by Teng et al. [29], (ii) Wu and Wei’s general model for FRP-confined NAC [30], (iii) Wang et al.’s model for FRP-confined CAC [7], and (iv) the model of Zhou et al. for FRP-confined lightweight aggregate concrete [31].

**Figure 11:**
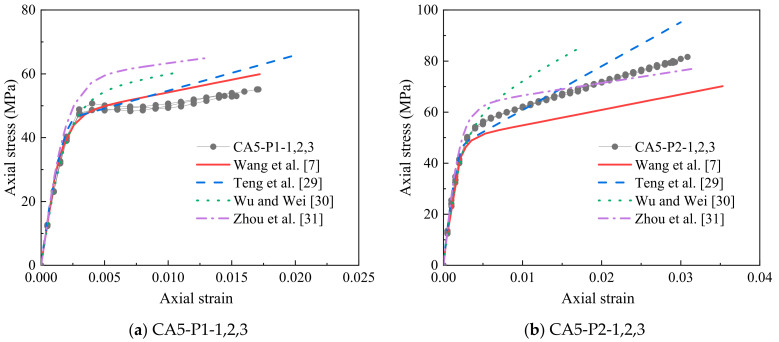

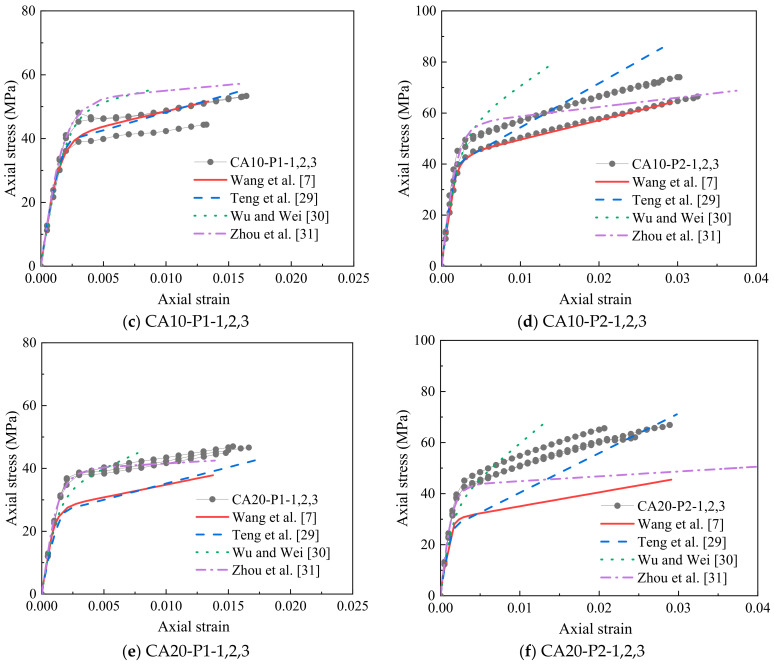
Comparison of the stress–strain models.

Figure 11 shows the comparison of the measured axial stress–strain curves and the results from the aforementioned available four models. These models cannot well anticipate the axial stress–strain relationship of FRP-confined CAC. The model of Teng et al. [29] can predict the ultimate axial strain well, whereas both the ultimate strength and secondary stiffness of the curves are generally overestimated. The model of Wu and Wei [30] greatly underestimates the ultimate axial strain while vastly overestimating the secondary stiffness. The above two models are applicable for NAC, whose properties are different from the properties of CAC. The model of Zhou et al. [31] exhibits an overestimation of the ultimate stress and underestimation of the ultimate axial strain for 1-ply FRP-confined CAC but has the opposite trend for 2-ply FRP-confined CAC because Zhou et al.’s model [31] is applicable for lightweight aggregate concrete, which is different from CAC. The model of Wang et al. [7] greatly underestimates the ultimate strength, as it was regressed from the test results of three specimens with only one concrete strength grade. In addition to the strength and ultimate strain, there is an evident difference in the transition segment (the value of *f*_0_) between the measured and predicted results. The larger the coral aggregate size is, the greater the underprediction of *f*_0,_ due to the more pronounced brittleness of CAC compared to the brittleness of NAC, especially for CAC with a larger aggregate size. Macroscopic cracks appeared for unconfined CAC when the peak stress was reached (Figure 3a), and the macroscopic cracks were greatly restricted by the external FRP jacket. It was reported that the *f*_0_/*f_co_* for the FRP-confined high-strength concrete was greater than that of the FRP-confined normal-strength concrete [32]. The more brittle failure mode of high-strength concrete in terms of fewer but larger cracks accounts for this test phenomenon.

**Figure 12:**
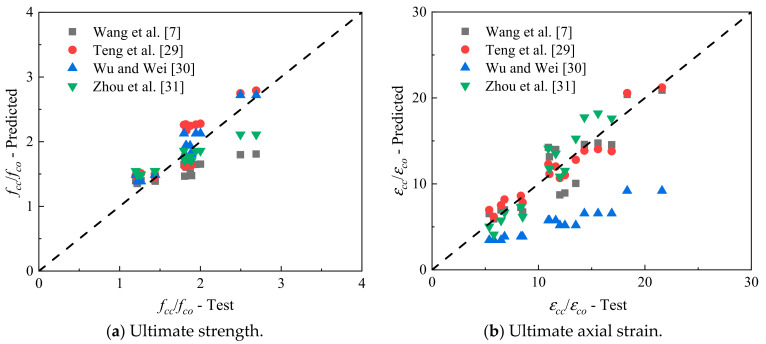
Performance of ultimate condition models.

Figure 12 compares the two ratios of *f_cc_*/*f_co_* and *ε_cc_/ε_co_* of FRP-confined CAC between the measured results and the results predicted from the existing four models. Table 7 lists the average absolute error (*AAE*) of each model, which is calculated by:

(2) $$AAE=\frac{\sum \left(\left|Expe.-Theo.\right|/Expe.\right)}{n}$$

where *n* is the total number of specimens. Table 7 and Figure 11 show that the model of Wu and Wei [30] performs best in the ultimate strength prediction, and Teng et al.’s model [29] gives the most accurate prediction of the ultimate axial strain.

## 5. Performance of the Modified Stress–Strain Model

**Figure 13:**
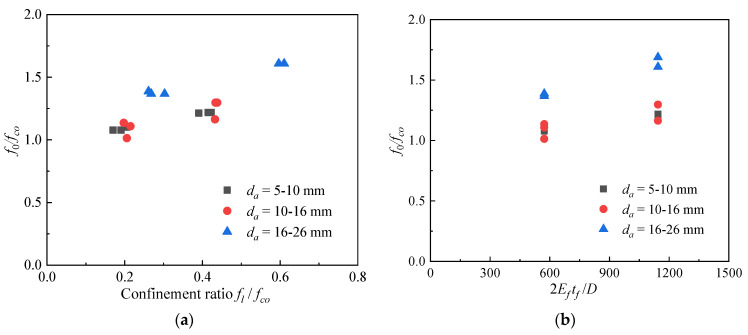
The relationship between the *f*_0_/*f_co_* and *f_l_*/*f_co_* and confinement stiffness (2*E_f_t_f_/D*). (**a**) Relationship between the *f*_0_/*f_co_* and *f_l_*/*f_co_*. (**b**) Relationship between the *f*_0_/*f_co_* and 2*E_f_t_f_/D*.

From the previous analysis, we can infer that a modification should be carried out based on the existing stress–strain models to reflect the mechanical properties of FRP-confined CAC well. Because the properties of CAC are closer to the properties of lightweight aggregate concrete, the stress–strain expression employed by Wang et al. [7] and Zhou et al. [31] was used as the benchmark, which is given by:

(3) $$\sigma_{c}=\left[\left(E_{1}\varepsilon_{n}-f_{0}\right)e^{-\frac{\varepsilon_{c}}{\varepsilon_{n}}}+f_{0}+E_{2}\varepsilon_{c}\right]\left(1-e^{-\frac{\varepsilon_{c}}{\varepsilon_{n}}}\right)$$

where *σ_c_* = the axial stress of the concrete, *ε_c_* = the axial strain of the concrete, *E*_1_ = the slope of the first branch of the stress–strain curve, *ε_n_* = *nε_o__,_* where *ε_o_* = *f*_0_/*E*_1_, and *n* is the curve shape parameter that controls the curvature of the transition zone, which satisfies 0 < *n* < 1. According to the previous analysis, the model of *f_cc_* proposed by Wu and Wei [30] and the model of *ε_cc_* proposed by Wang et al. [7] were used to predict the ultimate state of the FRP-confined CAC, which is given in Table 7. In addition to the *f_cc_* and *ε_cc_*, there are four parameters (*E*_1_*, f*_0_, *E*_2_, *n*) to be determined in Eq. (3) to develop the stress–strain model of the FRP-confined CAC. *E*_1_, *E*_2_, and *n* can be determined accordingly as in Zhou et al. [31] (*E*_1_ = *E_c_*, *n* = 0.5, *E*_2_ = (*f_cc_*–*f*_0_)/*ε_cc_*). Through the comparison between the measured stress–strain curves and the existing models in Figure 11, none of the four models performed well in predicting the *f*_0_. Therefore, it is necessary to propose a new model for the *f*_0_ of FRP-confined CAC. Through careful examination of the test results, the value of *f*_0_/*f_co_* was found to be proportional to *f_l_*/*f_co_* and the confinement stiffness (2*E_f_t_f_/D*), as shown in Figure 13. Through regression of the test results, the following model is thus proposed:

(4) $$\frac{f_{0}}{f_{co}}=1+2.235{\left(\frac{f_{l}}{f_{co}}\right)}^{0.87}-0.0007\frac{2E_{f}t_{f}}{D}$$

**Figure 14:**
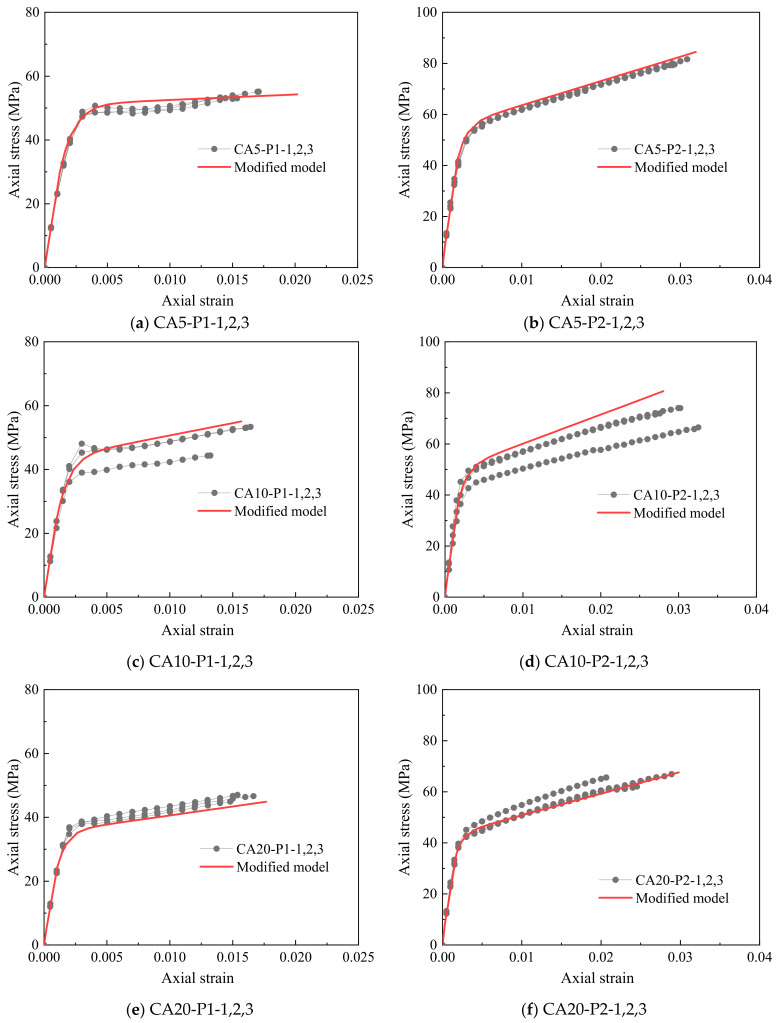
Performance of the modified model.

As seen in Figure 14, the modified model was much more accurate than these available models in representing the stress–strain response of the FRP-confined CAC with various aggregate sizes because the suggested modified model properly considers the effect of the unconfined compressive strength of CAC resulting from different coral aggregate sizes. Unfortunately, because of the delay in expansion and the utilization of the bilinear stress–strain relationship of CAC, the slight stress drop following the transition zone cannot be captured by the modified model. However, this slight stress drop does not affect the accurate representation of the ultimate condition by using the modified model.

## 6. Practical Implications of the Present Study

With the increasing demand for the construction of global island projects, coral concrete is one of the hot research topics in the field of civil engineering worldwide. China has regarded the strategy of marine power as the basic national policy. There are more than 200 islands and countless coral reefs and amounts of sea sand in the South China Sea. Using these locally available raw materials for preparing concrete is beneficial for reducing energy consumption and significantly avoiding harm to the environment during transportation. Although any non-renewable resource, including coral reefs and sea sand, unavoidably will face the problem of resource depletion, the planned and rational application of these raw materials will have a negligible negative impact on the local ecological environment. Currently, scarce work is available with respect to the optimized treatment of the coral aggregate in improving the mechanical behavior of CAC. The main attempt of the present work is to explore the influence of coral aggregate size on the axial compression characteristics of unconfined and FRP-confined CAC. A new stress–strain model is also proposed for the sustainable design of eco-friendly CAC members. The results from the present test indicate that a smaller coral aggregate size is more desirable for the enhancement of the properties of both unconfined and confined CAC. Since the tailoring of the size of coral aggregate is easily available, the research findings of the present study are conducive to further promoting the low-carbon sustainable application of coral aggregate in remote island construction.

## 7. Conclusions

This study investigated the effect of coral aggregate size on the axial compressive properties of the FRP-confined CAC. A group of FRP-confined CAC cylinders with test parameters of aggregate size and FRP thickness was prepared and tested under uniaxial compression. The experimental results, including the failure modes, axial stress versus strain response, hoop strain distributions, and ultimate conditions are discussed in a detailed manner. Several conclusions are summarized as follows:

(1) Owing to the low strength of the coral aggregate, the failure mode was rather brittle for the unconfined CAC. The aggregate size had a pronounced influence on the mechanical properties of the CAC. The larger the size of the coral aggregate was, the lower the strength and the smaller the ultimate axial strain of the CAC. Under the same mixture proportions, when the coral aggregate size increased from 5–10 mm to 16–26 mm, a 43.9% and 51.9% reduction of the stress and the ultimate strain were observed, respectively.
(2) The strength and ductility of CAC could be enhanced significantly by using external FRP confinement. The coral aggregate size had a pronounced effect on the dilation property of the CAC. The CAC with a smaller aggregate size dilated more uniformly than the CAC with the same FRP thickness but a larger aggregate size, suggesting a higher FRP confinement efficiency. When the coral aggregate size decreased from 16–26 mm to 5–10 mm, the average values of the *ε_h,rup_*/*ε_fu_* improved by 19.0% and 18.9%, respectively, for the columns confined by one and two-ply FRP jackets.
(3) The ultimate axial stress of the FRP-confined CAC was inversely proportional to the coral aggregate size. When the coral aggregate size increased from 5–10 mm to 16–26 mm, the average peak stress decreased by 42.9%, 14.2%, and 19.1%, respectively, for the columns without confinement, confined by one-, and two-ply FRP jackets. However, the coral aggregate size had little influence on the strength and ultimate axial strain enhancements as the confinement ratio increased.
(4) None of the existing models can capture the stress–strain behavior of FRP-confined CAC well. A modified stress–strain model is subsequently suggested for FRP-confined CAC with the careful consideration of the models of the ultimate condition as well as the model of the stress at the transition segment (the value of *f*_0_). The modified model provided a reasonable prediction of the stress–strain curve of the FRP-confined CAC.
(5) A smaller coral aggregate size is more desirable for the property enhancement of both unconfined and confined CAC. Since the tailoring of the size of coral aggregate is easily available, the research findings of the present study are conducive to further promoting the low-carbon sustainable application of coral aggregate in remote island construction.

## Figures and Tables

**Table 1:** Detailed information of all tested column specimens.

Specimen ID	*d_a_* (mm)	FRP Layers	*f_co_* (MPa)	*e_co_* (%)	*f_cc_* (MPa)	*e_cc_* (%)	*e_h_*_,rup_ (%)	*f_l_* (MPa)	*E_c_* (MPa)	*E*_2_ (MPa)	*f*_0_ (MPa)	*f_cc_/f_co_*	*e_cc_/e_co_*
CA5-P0-1	5–10	0	43.1	0.27					25,206				
CA5-P0-2			44.1	0.26					24,673				
CA5-P0-3			-	-					-				
CA10-P0-1	10–16		37.4	0.18					23,698				
CA10-P0-2			37.3	0.20					24,230				
CA10-P0-3			36.3	0.20					23,210				
CA20-P0-1	16–26		24.3	0.15					25,304				
CA20-P0-2			24.6	0.11					25,118				
CA20-P0-3			25.8	0.14					23,163				
CA5-P1-1	5–10	1	43.6	0.27	53.3	1.43	1.44	8.2	23,136	483	45.7	1.22	5.38
CA5-P1-2			43.6	0.27	55.1	1.72	1.57	9.0	23,131	562	45.4	1.26	6.49
CA5-P1-3			43.6	0.27	53.1	1.54	1.41	8.1	22,834	223	48.4	1.22	5.81
CA5-P2-1		2	43.6	0.27	79.6	2.93	1.50	17.2	26,666	1025	50.7	1.83	11.03
CA5-P2-2			43.6	0.27	81.6	3.09	1.57	17.9	23,798	1014	51.3	1.87	11.62
CA5-P2-3			43.6	0.27	79.3	2.90	1.58	18.1	23,403	1030	50.8	1.82	10.93
CA10-P1-1	10–16	1	37.0	0.19	53.1	1.61	1.48	8.5	24,514	486	44.4	1.44	8.35
CA10-P1-2			37.0	0.19	44.4	1.31	1.50	8.6	21,954	482	37.7	1.20	6.81
CA10-P1-3			37.0	0.19	53.3	1.64	1.32	7.6	24,079	615	43.0	1.44	8.52
CA10-P2-1		2	37.0	0.19	74.0	3.01	1.46	16.7	24,966	966	46.7	2.00	15.59
CA10-P2-2			37.0	0.19	71.9	2.76	1.44	16.5	27,558	908	47.8	1.94	14.32
CA10-P2-3			37.0	0.19	66.5	3.25	1.41	16.2	21,193	758	42.3	1.80	16.88
CA20-P1-1	16–26	1	24.9	0.13	47.0	1.54	1.12	6.4	24,470	748	35.9	1.89	12.49
CA20-P1-2			24.9	0.13	46.6	1.66	1.35	7.7	22,521	622	36.3	1.88	13.51
CA20-P1-3			24.9	0.13	44.9	1.48	1.15	6.6	22,986	604	35.7	1.81	12.01
CA20-P2-1		2	24.9	0.13	66.9	2.89	1.38	15.8	23,123	939	40.9	2.69	21.61
CA20-P2-2			24.9	0.13	65.7	2.07	-	-	25,209	1167	42.6	2.64	15.50
CA20-P2-3			24.9	0.13	62.1	2.45	1.30	14.8	23,955	988	40.4	2.50	18.34

“-” Data is missing due to misoperation.

**Table 2:** Chemical composition of seawater.

K^+^ (mg/L)	Na^+^ (mg/L)	Ca^2+^ (mg/L)	Mg^2+^ (mg/L)	CO_3_^2−^ (mg/L)	SO_4_^2−^ (mg/L)	Cl^−^ (mg/L)	Br^−^ (mg/L)
282	1650	276	1080	72	2110	14,900	53

**Table 3:** Physical properties and chemical composition of sea sand.

Apparent Density (kg/m^3^)	Bulk Density (kg/m^3^)	Mud Content (%)	Seashells (%)	Cl^–^ (%)	SO_4_^2–^ (%)	Fineness Modulus
2520	1430	1.3	0.4	0.01	0.3	2.2

**Table 4:** Physical properties of coral coarse aggregate.

Coral Aggregate Size (mm)	Apparent Density (kg/m^3^)	Saturated Water Absorption (%)	Crushing Index (%)
5–10	2472	11.67	-
10–16	2371	11.86	35.46
16–26	2288	12.19	-

**Table 5:** Mixture proportions of CAC.

ID	Cement (kg/m^3^)	Sea Water (kg/m^3^)	Sea Sand (kg/m^3^)	Coral Aggregate (kg/m^3^)	Superplasticizer (kg/m^3^)	Concrete Strength in 28 Days (MPa)
CA5	616	300	635	729	2.59	33.5
CA10	616	300	635	729	1.40	32.9
CA15	616	300	635	729	0.98	27.2

**Table 6:** Mechanical properties of CFRP.

CFRP	Type	Thickness *t_f_* (mm)	Ultimate Strength *f_frp_* (Gpa)	Ultimate Strain *ε_fu_* (%)	Elastic Modulus *E_f_* (Gpa)
Manufacturer	UT70-30	0.167	3.4	1.48	230
coupon tests			3.9	1.53	257

**Table 7:** Existing models for ultimate condition.

Reference	Ultimate Strength Models	*AAE*	Ultimate Strain Models	*AAE*
Wang at al. [7]	$\frac{f_{cc}}{f_{co}}=1+2.11{\left(0.375\frac{f_{l}}{f_{co}}\right)}^{0.65}$	0.149	$\frac{\varepsilon_{cc}}{\varepsilon_{co}}=1.5+36.2{\left(\rho_{K}\right)}^{1.17}{\left(\rho_{\varepsilon }\right)}^{1.15}$	0.149
Teng et al. [29]	$\frac{f_{cc}}{f_{co}}=1+3.5\left(\rho_{K}-0.01\right)\rho_{\varepsilon }$	0.148	$\frac{\varepsilon_{cc}}{\varepsilon_{co}}=1.75+6.5{\left(\rho_{K}\right)}^{0.8}{\left(\rho_{\varepsilon }\right)}^{1.45}$	0.102
Wu and Wei [30]	$\frac{f_{cc}}{f_{co}}=0.75+2.7{\left(\frac{f_{lu}}{f_{co}}\right)}^{0.9}$	0.080	$\frac{\varepsilon_{cc}}{\varepsilon_{co}}=1.75+140\left(\frac{f_{lu}}{f_{co}}\right)\varepsilon_{fu}^{0.6}$	0.516
Zhou at al. [31]	$\frac{f_{cc}}{f_{co}}=1+2.11{\left(\frac{0.53f_{lu}}{f_{co}}\right)}^{0.65}$	0.113	$\frac{\varepsilon_{cc}}{\varepsilon_{co}}=1.5+5.24{\left(\rho_{K}\right)}^{1.45}{\left(\rho_{\varepsilon }\right)}^{2.63}$	0.239

Note: *ρ_K_* is the confinement stiffness ratio, *ρ_ε_* is the strain ratio, and *f_lu_* is the confinement strength, which are calculated as follows:$f_{l}=\frac{2E_{f}t_{f}\varepsilon_{h,\mathrm{rup}}}{D}$, $f_{lu}=\frac{2E_{f}t_{f}\varepsilon_{fu}}{D}$, $\rho_{K}=\frac{2E_{f}t_{f}}{\left(f_{co}/\varepsilon_{co}\right)D}$, $\rho_{\varepsilon }=\frac{\varepsilon_{h,\mathrm{rup}}}{\varepsilon_{co}}$.

## Data Availability

All data, models, or codes generated or used during the study are available upon request.
